# A bacterial culture collection from stable flies and manure relevant to bovine mastitis

**DOI:** 10.1128/mra.00805-25

**Published:** 2025-10-20

**Authors:** Andrew J. Sommer, Travis K. Worley, Julia E. Kettner, Kerri L. Coon

**Affiliations:** 1 Department of Bacteriology, University of Wisconsin-Madison 5228, Madison, Wisconsin, USA; Indiana University Bloomington, Bloomington, Indiana, USA

**Keywords:** insect pests, bacterial isolates, mastitis pathogens, antimicrobial resistance, host-vector interactions, environmental reservoirs, comparative genomics, microbiota diversity, veterinary microbiology, culture collection

## Abstract

We present a collection of over 1,100 bacterial isolates from hematophagous *Stomoxys* flies and cow manure. Genome assemblies and antimicrobial susceptibility data are available for a subset of isolates. This resource supports studies of bacterial ecology, host-vector interactions, and potential roles in bovine mastitis.

## ANNOUNCEMENT

Stable flies of the genus *Stomoxys* (Diptera: Muscidae) are common blood-feeding pests of dairy cows and other livestock. They have long been recognized as mechanical vectors of manure-borne bacterial pathogens and antimicrobial resistance genes (1–4). More recently, these flies have also been implicated in the transmission of mastitis-causing bacteria in dairy environments, particularly through their frequent contact with manure and contaminated surfaces (2–4). Despite these associations, the phylogenetic and functional diversity of their microbiota remain poorly characterized.

To address this gap, we present a bacterial culture collection derived from *Stomoxys* flies, along with the publicly available draft genome assemblies of bacteria isolated from these insects, as detailed in a companion article to this announcement (5). This resource enables computational and experimental investigations into fly-bacteria interactions and their implications for veterinary health, with particular relevance to the ecology and transmission of environmental mastitis pathogens.

Isolates were collected from flies, manure, and mastitic cows housed at two interconnected dairy farms in Wisconsin, USA. *Stomoxys* flies were captured using adhesive alsynite fiberglass traps (Olson Products, Medina, OH, USA), and manure samples were obtained from both scraper systems and cattle pens between July and September 2021. Flies were washed in PBS-T (1× PBS with 0.01% Tween 80), and exogenous bacteria were recovered from the wash solution following centrifugation. Endogenous bacteria were isolated from fly homogenates prepared by bead-beating pooled flies with 3 × 5 mm^2^ stainless steel beads. Field samples—including diluted feces, fly homogenates, and fly washes—were plated on various nutrient agars, including MacConkey, Sorbitol-MacConkey, Mannitol Salt, Brain Heart Infusion, Tryptic Soy, blood, and Eosin Methylene Blue (EMB). Strains from mastitic cows were obtained from archived samples maintained by the Wisconsin Veterinary Diagnostic Laboratory (Madison, Wisconsin, USA). Detailed sampling and isolation procedures are described in previous studies (2–5).

The collection comprises 909 fly-derived, 206 manure-derived, and 12 mastitic cow-derived taxonomically identified isolates, including clinically relevant taxa within the *Bacillales*, *Enterobacterales*, and *Pseudomonadales* (Table 1). Species-level identification is available for the 296 isolates with draft genome assemblies. The remaining isolates were identified via Sanger sequencing of the 16S rRNA gene, phenotypic methods (e.g., colony morphology on EMB agar, where *Escherichia coli* typically produces a metallic green sheen), or PCR amplification using genus-specific primers.

**TABLE 1:** Collection composition and sequencing overview

Key taxa in the collection^*a*^										
Taxon			Fly isolates			Manure isolates			Mastitis isolates	
			Total	WGS		Total	WGS		Total	WGS
*Bacillales*	*Bacillus* spp.		99	4		4	4		0	0
	*Staphylococcaceae*		226	127		78	17		2	1
*Enterobacterales*	*Erwiniaceae*		79	0		0	0		0	0
	*Escherichia* spp.		66	36		53	28		1	1
	*Klebsiella* spp.		55	24		31	20		7	6
	Other *Enterobacteriaceae*		178	10		26	7		0	0
	*Yersiniaceae*		37	2		0	0		0	0
*Pseudomonadales*	*Acinetobacter* spp.		41	5		6	4		0	0
	*Pseudomonas* spp.		40	0		0	0		0	0

Key sequencing metrics			
Metric	Minimum	Maximum	Median
Total length	2,302,962 bp	5,849,702 bp	3,241,861 bp
Number of contigs	21	574	95
GC content	31.4%	59.9%	39.1%
N50	37,008	2,777,308	285,280
L50	1	28	5
Complete BUSCOs^*b*^	118	124	124
CheckM completeness^*c*^	95.91%	100.00%	99.45%
CheckM contamination^*c*^	0.00%	4.38%	0.74%

^*a*^ The table highlights select taxonomic groups of interest. While not exhaustive, it represents the majority of the bacterial taxa present in the collection.

^*b*^ Genomes encode a maximum of 124 benchmarking universal single-copy orthologs (BUSCOs).

^*c*^ CheckM metrics determined using a full reference tree lineage.

DNA extractions for strains selected for whole genome sequencing were performed using either the NucleoSpin Tissue kit (Macherey-Nagel, Nordrhein-Westfalen, Germany) for samples processed by SeqCoast Genomics (Portsmouth, NH, USA) or the DNeasy Blood & Tissue Kit (Qiagen, Hilden, Germany) for samples processed by the UW-Madison Biotechnology Center, following standard manufacturer protocols. Genomic DNA from 190 isolates sent to SeqCoast Genomics was prepared using the Illumina DNA Prep Tagmentation Kit and sequenced on the NextSeq2000 platform, generating 2 × 150 bp paired-end reads with an average of 1.3 million reads per sample. DNA from 106 isolates sent to the UW-Madison Biotechnology Center was prepared using the Celero EZ DNA-Seq Prep Kit (Tecan Genomics, Männedorf, Switzerland) and sequenced on the Illumina NovaSeq6000 platform, producing 2 × 150 bp paired-end reads with approximately 1 million reads per sample.

Sequence read trimming was performed using DRAGEN v3.10.12 for SeqCoast Genomics samples and Trimmomatic v0.39 (6) for UW-Madison samples. Draft genome assemblies were generated using SPAdes v3.15.5 and annotated with PROKKA v 1.13 using the “rfam” setting (7, 8). Quality control was conducted throughout the workflow. Sequencing data quality was assessed using FastQC v0.11.9, QUAST v5.2.0, CheckM v1.0.18, and BUSCO v 5.7.1 (9–12). Summary statistics for sequence quality metrics are provided in Table 1.

Isolates from the *Staphylococcaceae* and *Enterobacteriaceae* were screened for antimicrobial susceptibility using CLSI disk diffusion guidelines (4). Notably, the collection includes fly-derived strains of *E. coli*, *Klebsiella pneumoniae*, and *Enterobacter* spp. carrying extended-spectrum beta-lactamase (ESBL) genes conferring resistance to ceftiofur—a key antibiotic used to treat bovine mastitis and other infections on U.S. dairy farms (13). These findings underscore the potential role of flies in the environmental dissemination of mastitis-associated pathogens and resistance determinants.

We encourage the scientific community to use this collection to investigate host-microbe interactions, bacterial dispersal by muscid flies, and the environmental reservoirs of mastitis pathogens. Comparative genomic analyses have already revealed shared strains and plasmids between flies and manure (5). In addition, the collection supports colonization and related studies using field-derived strains, which often retain genotypic and phenotypic traits absent in domesticated laboratory strains—traits that are essential for capturing the complexity of natural microbial associations relevant to mastitis ecology.

## Data Availability

Bacterial isolates and associated metadata are available upon request from the corresponding author. Raw Illumina reads and genome assemblies for sequenced isolates are publicly accessible via NCBI under BioProject ID PRJNA1216036.

## References

[1] Baldacchino F, Muenworn V, Desquesnes M, Desoli F, Charoenviriyaphap T, Duvallet G. 2013. Transmission of pathogens by Stomoxys flies (Diptera, Muscidae): a review. Parasite 20:26. doi:10.1051/parasite/2013026 23985165 PMC3756335

[2] Sommer AJ, Kettner JE, Coon KL. 2024. Stable flies are bona fide carriers of mastitis-associated bacteria. mSphere 9:e0033624. doi:10.1128/msphere.00336-24 38920390 PMC11288000

[3] Sommer AJ, Deblois CL, Tu ADJ, Suen G, Coon KL. 2025. Opportunistic pathogens are prevalent across the culturable exogenous and endogenous microbiota of stable flies captured at a dairy facility. Vet Res 56:40. doi:10.1186/s13567-025-01458-3 39934928 PMC11817017

[4] Sommer AJ, Kettner JE, Worley TK, Petrick J, Haynie C, Coon KL. 2025. Prevalence of antimicrobial resistance phenotypes and genes in stable fly- and manure-derived bacterial isolates from clinically relevant taxa in dairy settings. J Appl Microbiol 136:lxaf025. doi:10.1093/jambio/lxaf025 39886877

[5] Sommer AJ, Worley TK, Sapountzis P, Coon KL. 2025. Phylogenetic intermixing reveals stable fly-mediated circulation of mastitis-associated bacteria in dairy settings. mSystems 10:e00215–25. doi:10.1128/msystems.00215-25 40748061 PMC12455971

[6] Bolger AM, Lohse M, Usadel B. 2014. Trimmomatic: a flexible trimmer for Illumina sequence data. Bioinformatics 30:2114–2120. doi:10.1093/bioinformatics/btu170 24695404 PMC4103590

[7] Prjibelski A, Antipov D, Meleshko D, Lapidus A, Korobeynikov A. 2020. Using SPAdes De Novo assembler. CP in Bioinformatics 70:e102. doi:10.1002/cpbi.102 32559359

[8] Seemann T. 2014. Prokka: rapid prokaryotic genome annotation. Bioinformatics 30:2068–2069. doi:10.1093/bioinformatics/btu153 24642063

[9] Simon A. 2010. Internet. FastQC: a quality control tool for high throughput sequence data. Available from: http://www.bioinformatics.babraham.ac.uk/projects/fastqc

[10] Gurevich A, Saveliev V, Vyahhi N, Tesler G. 2013. QUAST: quality assessment tool for genome assemblies. Bioinformatics 29:1072–1075. doi:10.1093/bioinformatics/btt086 23422339 PMC3624806

[11] Simão FA, Waterhouse RM, Ioannidis P, Kriventseva EV, Zdobnov EM. 2015. BUSCO: assessing genome assembly and annotation completeness with single-copy orthologs. Bioinformatics 31:3210–3212. doi:10.1093/bioinformatics/btv351 26059717

[12] Parks DH, Imelfort M, Skennerton CT, Hugenholtz P, Tyson GW. 2015. CheckM: assessing the quality of microbial genomes recovered from isolates, single cells, and metagenomes. Genome Res 25:1043–1055. doi:10.1101/gr.186072.114 25977477 PMC4484387

[13] Gonçalves JL, de Campos JL, Steinberger AJ, Safdar N, Kates A, Sethi A, Shutske J, Suen G, Goldberg T, Cue RI, Ruegg PL. 2022. Incidence and treatments of bovine mastitis and other diseases on 37 dairy farms in Wisconsin. Pathogens 11:1282. doi:10.3390/pathogens11111282 36365033 PMC9698317

